# Genetic aspects of lactase deficiency in indigenous populations of Siberia

**DOI:** 10.18699/vjgb-24-72

**Published:** 2024-10

**Authors:** B.A. Malyarchuk

**Affiliations:** Institute of Biological Problems of the North of the Far Eastern Branch of the Russian Academy of Sciences, Magadan, Russia

**Keywords:** genetic polymorphism, lactase persistence, MCM6 gene, LCT gene, human populations, Siberia, archaic variants of polymorphism, генетический полиморфизм, персистенция лактазы, ген MCM6, ген LCT, популяции человека, Сибирь, архаичные варианты полиморфизма

## Abstract

The ability to metabolize lactose in adulthood is associated with the persistence of lactase enzyme activity. In European populations, lactase persistence is determined mainly by the presence of the rs4988235-T variant in the MCM6 gene, which increases the expression of the LCT gene, encoding lactase. The highest rates of lactase persistence are characteristic of Europeans, and the lowest rates are found in East Asian populations. Analysis of published data on the distribution of the hypolactasia-associated variant rs4988235-C in the populations of Central Asia and Siberia showed that the frequency of this variant increases in the northeastern direction. The frequency of this allele is 87 % in Central Asia, 90.6 % in Southern Siberia, and 92.9 % in Northeastern Siberia. Consequently, the ability of the population to metabolize lactose decreases in the same geographical direction. The analysis of paleogenomic data has shown that the higher frequency of the rs4988235-T allele in populations of Central Asia and Southern Siberia is associated with the eastward spread of ancient populations of the Eastern European steppes, starting from the Bronze Age. The results of polymorphism analysis of exons and adjacent introns of the MCM6 and LCT genes in indigenous populations of Siberia indicate the possibility that polymorphic variants may potentially be related to lactose metabolism exist in East Asian populations. In East Asian populations, including Siberian ethnic groups, a ~26.5 thousand nucleotide pairs long region of the MCM6 gene, including a combination of the rs4988285-A, rs2070069-G, rs3087353-T, and rs2070068-A alleles, was found. The rs4988285 and rs2070069 loci are located in the enhancer region that regulates the activity of the LCT gene. Analysis of paleogenomic sequences showed that the genomes of Denisovans and Neanderthals are characterized by the above combination of alleles of the MCM6 gene. Thus, the haplotype discovered appears to be archaic. It could have been inherited from a common ancestor of modern humans, Neanderthals, and Denisovans, or it could have been acquired by hybridization with Denisovans or Neanderthals. The data obtained indicate a possible functional significance of archaic variants of the MCM6 gene.

## Abstract (ru)

Способность метаболизировать лактозу во взрослом состоянии связана с сохранением активности фермента лактазы. В европейских популяциях персистенция лактазы детерминируется главным образом наличием варианта rs4988235-T в гене MCM6, который увеличивает экспрессию гена LCT, кодирующего лактазу. Наиболее высокие показатели персистенции лактазы характерны для европейцев, а самые низкие – для населения Восточной Азии. Анализ опубликованных данных о распределении варианта rs4988235-C, связанного с гиполактазией, у населения Центральной Азии и Сибири выявил, что частота этого варианта увеличивается в северо-восточном направлении. В Центральной Азии частота этого аллеля составляет 87 %, на юге Сибири – 90.6 % и на северо-востоке Сибири – 92.9 %. Соответственно, в таком же географическом направлении убывает способность населения метаболизировать лактозу. Анализ палеогеномных данных показал, что более высокая частота аллеля rs4988235-T в популяциях Центральной Азии и Южной Сибири связана с распространением на восток древнего населения восточноевропейских степей начиная с эпохи бронзового века. Результаты анализа полиморфизма экзонов и прилегающих к ним интронов генов MCM6 и LCT у коренного населения Сибири свидетельствуют о возможности существования в восточноазиатских популяциях вариантов полиморфизма, потенциально связанных с метаболизмом лактозы. В популяциях Восточной Азии, в том числе в сибирских этнических группах, обнаружен участок гена MCM6 длиной ~26.5 тыс. пар нуклеотидов, включающий комбинацию аллелей rs4988285-A, rs2070069-G, rs3087353-T, rs2070068-A. Локусы rs4988285 и rs2070069 находятся в области энхансера, регулирующего активность гена LCT. Анализ палеогеномных последовательностей показал, что указанной выше комбинацией аллелей гена MCM6 характеризуются геномы денисовцев и неандертальцев. Таким образом, обнаруженный гаплотип, по всей видимости, является архаичным. Он мог быть унаследован от общего предка современных людей, неандертальцев и денисовцев, или же был приобретен в результате гибридизации с денисовцами или неандертальцами. Полученные данные свидетельствуют о возможной функциональной значимости архаичных вариантов полиморфизма гена MCM6

## Introduction

Lactose (milk sugar) is the main disaccharide in the milk of various mammals and its hydrolysis requires the enzyme lactase, encoded by the LCT gene, which is predominantly expressed in the small intestine. Lactase activity declines during ontogenesis, which can lead to difficulties digesting lactose in many adults (Ségurel, Bon, 2017). Primary hypolactasia (OMIM: 223100) is characterized by a range of symptoms (bloating, nausea, diarrhoea) after ingestion of milk and dairy products. However, ethnoregional populations around the world have been found to differ in their ability to metabolize lactose (Evershed et al., 2022). It is thought that this ability, or lactase persistence (LP), is inherited. One of the most important genetic polymorphisms that have been linked to LP is the T variant at the rs4988235 locus of the MCM6 gene, which regulates the expression of the LCT gene (Enattah et al., 2002; Olds, Sibley, 2003; Troelsen et al., 2003). Although this genetic variant is about 14,000 nucleotide pairs away from the LCT gene (which is why it is often called –13910*T), it is responsible for increasing the enzymatic activity of lactase, which breaks down lactose into glucose and galactose molecules.

It would appear that the lowest LP values are characteristic of East Asian populations, while the highest are found in Europeans (Liebert et al., 2017). This is due to the fact that, according to archaeological data, dairy farming may have emerged in the steppe zone of the North Caucasus and the Black Sea region around 4–5 thousand years ago (kya) (Scott et al., 2022). Paleogenomic data suggests that the frequency of the LP-associated variant rs4988235-T began to increase around 6 kya within the ancestral EHG and CHG genomic components characteristic of Eastern European and Caucasian hunter-gatherers, respectively (Segurel et al., 2020; Irving- Pease et al., 2024). The linkage between the polymorphism variants in the rs4988235 and rs1438307 loci was also revealed, and the increase in frequency of the rs1438307-T allele may have begun much earlier than previously thought, around 12 kya (Irving-Pease et al., 2024). With regard to the rs1438307-T variant, it has been suggested that it may have arisen as a consequence of the adaptation of ancient humans to starvation and exposure to pathogens; this is based on the observation that it is involved in the regulation of the body’s energy expenditure and the development of metabolic diseases (Evershed et al., 2022).

Despite the great interest of the genetic and medical communities in the genetic aspects of hypolactasia in human populations, many regions of the world remain poorly studied (Liebert et al., 2017; Anguita-Ruiz et al., 2020). The aim of this paper is to attempt to review the results of studies on the polymorphism of the LCT and MCM6 genes, which are directly related to lactase persistence, in indigenous populations of Siberia, one of the least studied regions

## Distribution of rs4988235 locus polymorphisms in modern and ancient North Asian populations

Genetic and epidemiological studies have indicated that in populations of the European part of Russia, primary hypolactasia is determined predominantly or exclusively by the rs4988235-C allele of the MCM6 gene (Borinskaya et al., 2006; Kovalenko et al., 2023), and accordingly, LP is defined by the rs4988235-T allele. However, in East Asian populations (including Siberian ones), this relationship is not as clearly evident – some populations (e. g., Buryats and Uyghurs) show very high (at the level of 95 %) frequency of the rs4988235- C variant, which is associated with a reduced prevalence of hypolactasia (Borinskaya et al., 2006; Sokolova et al., 2007). In this context, it has been proposed that the lower frequency of hypolactasia in some ethnic groups of Siberia and Central Asia may be associated with the presence of not only the rs4988235-T variant, but also some other genetic LP markers (Sokolova et al., 2007)

To date, it has been found that in addition to the rs4988235- T allele, several other genetic polymorphism variants that determine the ability to break down lactose are common in African and Middle Eastern ethnic groups, for example they include the rs41525747, rs41380347, rs145946881 and rs182549 loci of the MCM6 gene (Ingram et al., 2007; Tishkoff et al., 2007). However, data on the association between genetic polymorphisms and LP in East Asian populations is somewhat more conflicting. For instance, in Central Asian populations, a mixed sample of Tajiks and Uzbeks, as well as Kazakhs, showed that the rs4988235-T variant (with frequencies of 10 and 16.5 %, respectively) correlated quite well with the ability to digest lactose (11–30 % in Tajiks and Uzbeks, and 25–32 % in Kazakhs) (Heyer et al., 2011). It would appear that Tibetans, who have a long-standing tradition of consuming yak milk, also digest lactose at a level of around 30 %, but they appear to lack the rs4988235-T and rs182549-T polymorphism variants that are found in neighbouring populations in northern China at a frequency of 3.8 and 6.9 %, respectively (Xu et al., 2010; Peng et al., 2012). Tibetans have been found to have their own spectrum of alleles of the enhancer region of the MCM6 gene, which may be associated with LP, among which the –13838*A variant appears to predominate with a frequency of about 6.5 % (Peng et al., 2012)

A more detailed study of the genetic adaptation to milk consumption in Central Asian populations, distinguished by their economic patterns, has demonstrated that pastoralists (Kazakhs, Kyrgyz, Karakalpaks, Buryats, Mongolians and Altaians), whose diets rely heavily on dairy products, do not have a higher ability to metabolize lactose than farmers (Turkmens, Tajiks and Uzbeks), who have a higher prevalence of the rs4988235-T variant (Sequrel et al., 2020). The relatively low frequency (~10 %) of this genetic variant is also observed in ethnic groups in southern Siberia (Khakasians, Shorians and Tubalars) who lead a semi-nomadic lifestyle and are engaged in forestry and taiga hunting (Sequrel et al., 2020). The data indicate that the frequency of occurrence of the rs4988235-T variant in Central Asian and Siberian populations is not significantly influenced by economic structure or milk consumption levels.

Table 1 presents the distribution of the rs4988235-C variant in various indigenous populations from Northeast China, Central Asia and Siberia. As can be observed, the frequency of this variant in the populations varies from 70 to 100 %. However, when the samples are divided into three regional groups, there is an increase in the frequency of the rs4988235-C variant from the south to the northeast of Siberia (see the Figure).

**Table 1.**
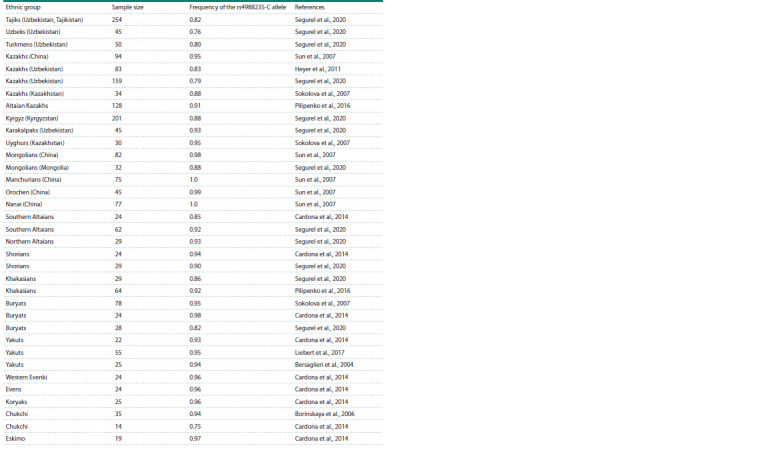
Frequency of the rs4988235-C allele in North Asian populations

**Fig. 1.**
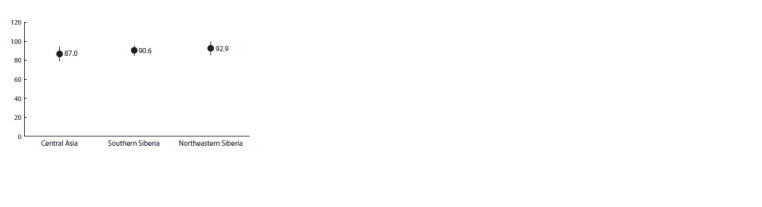
Distribution of the rs4988235-C variant in regional groups of Siberia and Central Asia. The average frequency of the genetic variant (in %) and the limits of the standard deviation of the frequency are shown.

The frequency of this allele is 87.0 ± 2.0 % in Central Asia, 90.6 ± 1.7 % in Southern Siberia and 92.9 ± 2.3 % in Northeastern Siberia. Consequently, the population’s capacity to metabolize lactose diminishes in a similar geographic direction. It is also noteworthy that the observed differences in the allele frequency of the rs4988235 locus are statistically significant only between the populations of Central Asia and Siberia (both its southern and northeastern parts; P < 10–5, Fisher’s exact test). Furthermore, the two Siberian populations do not differ from each other (P = 0.09). It is important to note that allele frequencies may vary in different samples within the same ethnic group. This is exemplified by the case of the Kazakhs, Buryats, and especially Chukchi (Table 1). In addition to random factors, which are particularly relevant in small sample sizes, admixture with individuals belonging to ethnic groups exhibiting a higher frequency of the rs4988235- T variant may contribute to the observed heterogeneity in allele frequencies.

This was clearly demonstrated in the study of the rs4988235 locus polymorphism in the Nenets, who have been reindeer herders for generations and who practically do not drink milk (Khabarova et al., 2012). It appears that this is primarily due to a high prevalence of lactose intolerance. The frequency of the rs4988235-C variant in the Nenets with all four grandparents of Nenets origin is 92.7 % (the frequency of the rs4988235- CC genotype is 90 %). Concurrently, the prevalence of the rs4988235-C allele among the Nenets with at least one relative of Nenets origin has declined to 73 % (Khabarova et al., 2012). The majority of interethnic marriages with Nenets involve Komi and North Russians, in whom the frequency of the rs4988235-T allele is 35–42 % (Khabarova et al., 2011). A certain decrease in hypolactasia among the populations of the northernmost regions of Europe and Siberia can be attributed to intermarriage contacts with immigrant populations of Eastern European origin, which commenced in the 17th century in conjunction with the expansion of the Russian pioneers and reached its peak during the Soviet period.

The duration and intensity of contacts with Eastern Europeans were evidently greater on the territory of Southwestern Siberia and Central Asia, taking into account the migrations of the populations of the Eastern European steppes during the Bronze Age. As previously stated, paleogenomic data indicates that the rs4988235-T mutation emerged approximately 6 kya in the ancient population of the northern Black Sea coast, and later this variant of the polymorphism spread throughout northern Eurasia, from Spain to Kazakhstan (Segurel et al., 2020; Irving-Pease et al., 2024). Furthermore, in Europe, the increased frequency of the rs4988235-T variant, which determines stable lactase activity required for milk digestion in adults, was favored by positive selection due to vitamin D deficiency at high latitudes and the need for increased calcium intake from milk (Kozlov, Vershubskaya, 2017).

A review of paleogenomic data in the AADR database (Allen Ancient DNA Resource, https://reich.hms.harvard.edu/) revealed that the first documented instances of the rs4988235- T allele were observed in ancient European populations, including those in Ukraine (~6 kya), Ireland (5.5 kya), and from 4.5 kya and later in Lithuania, Germany, Czech Republic, Estonia, and Russia. In East Asia, the rs4988235-T allele was first identified in an individual from the Botai archaeological culture in northern Kazakhstan (5.3 kya). In Central Asia (in the ancestors of the Kazakhs, Kyrgyz, Mongolians, Turkmens, Uzbeks, and Tajiks), the frequency of the rs4988235-T variant was 4.2 % between 0.5 and 5.3 kya. According to modern data (Table 1), its value is estimated to be approximately 13 %. It is hypothesized that this mutation was already prevalent in Central Asia with a frequency of approximately 5 % since the Iron Age (Segurel et al., 2020). Consequently, if this variant of genetic polymorphism was subject to strong selective pressure, it should have had sufficient time to reach high frequencies in modern populations. This is estimated by L. Segurel et al. (2020) to be 51 %. However, this was not the case, which leads to the reasonable conclusion that the rs4988235-T variant did not experience significant selective pressure in Central Asian populations, in contrast to Europeans and some populations in Africa and the Middle East (Segurel et al., 2020

The AADR database indicates that in the ancient populations of Siberia and the Urals (between 0.5 to 10 kya), the rs4988235-T variant was distributed with a frequency of 1.8 %, although only in the westernmost region of this territory. All cases of this allele were registered around 3.1–3.8 kya in representatives of the Karasuk (Southwestern Siberia) and Sintashta (Southern Urals) archaeological cultures. The mean frequency of the rs4988235-T variant in the contemporary indigenous population of Siberia is 0.8 % (Table 1). This suggests that, over the past 3,000 years, the frequency of the allele responsible for the enhancement of lactase enzymatic activity has remained unchanged in Siberian populations, despite changes in dietary habits and increased consumption of dairy products. This evidence indicates that in Siberian populations, the rs4988235-T allele behaves as a neutral variant of genetic polymorphism.

In light of the possibility of additional variants of the enhancer polymorphism of the MCM6 gene in the East Asian population, it is worth noting that this kind of screening was performed for several loci, including rs41525747, rs41380347, rs869051967, rs145946881, and rs182549 (Xu et al., 2010; Liebert et al., 2017; Anguita-Ruiz et al., 2020). The enhancer element of the LCT gene was also investigated in two indigenous populations of Southern Siberia – the Altaian Kazakhs and Khakasians (Pilipenko et al., 2016). Nevertheless, the frequencies of alleles potentially associated with LP were generally quite low. The sole exception to this is the rs182549 locus. In some East Asian populations, it has been reported that the rs182549-A allele is more informative than rs4988235-T. This is due to the observed occurrence of the rs182549-A allele in the absence of the rs4988235-T allele (Sun et al., 2007; Mattar et al., 2010; Xu et al., 2010). A similar conclusion has been reached for some populations of African, European, and West Asian origin (Bersaglieri et al., 2004; Coelho et al., 2005; Raz et al., 2013). However, this is at odds with the previous conclusion that a complete linkage disequilibrium exists between the rs4988235-T and rs182549-A alleles (Enattah et al., 2002; Troelsen et al., 2003), which included East Asian populations (Kato et al., 2018).

The data on the frequency of distribution of rs4988235-T and rs182549-A variants in ethnic groups of Siberia (Cardona et al., 2014) also indicate that these alleles are linked. The only exception is the Eskimo group, where the frequency of the rs182549-A allele exceeds that of the rs4988235-T allele (Table 2). Therefore, it is highly unlikely that the rs182549-A allele is responsible for maintaining lactase persistence in the indigenous Siberian population

**Table 2.**
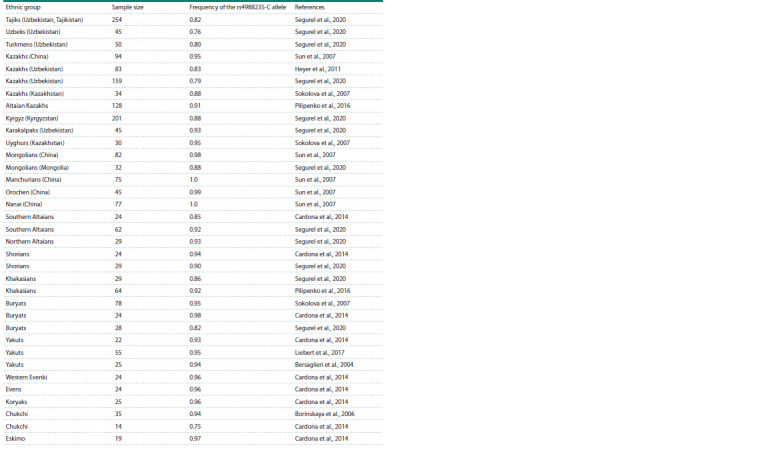
Frequencies of the rs4988235-T and rs182549-A alleles in Siberian populations (according to Cardona et al., 2014)

## Polymorphic variants (including archaic ones) of the LCT and MCM6 genes in indigenous Siberians

The results of the polymorphism analysis of exons and adjacent introns of the MCM6 and LCT genes in the indigenous populations of Siberia clearly indicate the existence of polymorphic variants potentially related to lactose metabolism in East Asian populations. Table 3 presents data on the distribution of LCT and MCM6 gene polymorphisms in 102 individuals from various regions of Siberia. These include the indigenous populations of Northeastern (Eskimo, Chukchi, Koryaks), Central (Evens, Evenki, Yakuts), Southern (Tuvinians, Shorians, Altaians, Buryats), and Western (Kets, Khanty, Mansi, Selkups, and Nenets) Siberia. The data were obtained from a full-genome variability study (Pagani et al., 2016). The LCT gene contains 21 polymorphic loci, while the MCM6 gene has seven. The majority of polymorphic variants identified in indigenous Siberians belong to alleles that are commonly found in both East Asian and European populations. Rare variants characteristic only of the East Asian population were found in the rs201668742, rs144864087, and rs3739021 loci. Similarly, variants characteristic only of Europeans were revealed in the rs34307240 locus.

**Table 3.**
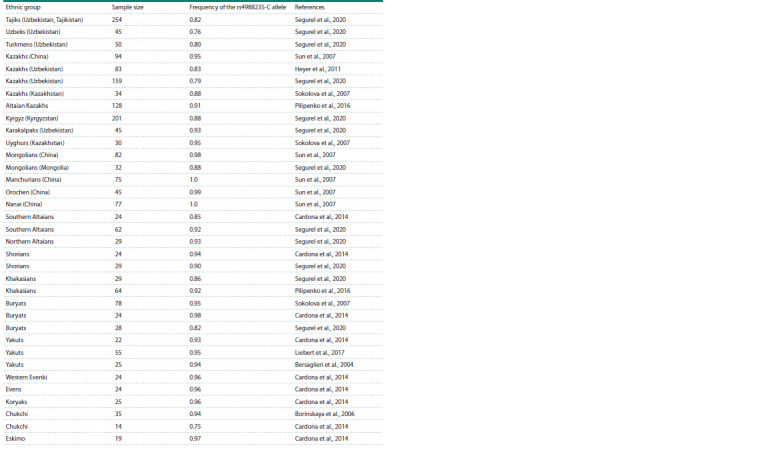
Polymorphic variants of exons and adjacent introns of the LCT and MCM6 genes and their frequency (in %) in Eurasian populations Notе. Designations: NES – Northeastern Siberia; CS – Central Siberia; SS – Southern Siberia; WS – Western Siberia; EAS – East Asia; EUR – Europe. For Siberian populations, frequencies are given according to Pagani et al. (2016), for East Asia and Europe, according to the dbSNP database

However, it is noteworthy that a group of polymorphic variants in the rs79023654 locus of the LCT gene and the rs4988285, rs2070069, rs3087353, and rs2070068 loci of the MCM6 gene (highlighted in bold in Table 3) warrant further investigation. The rs79023654, rs4988285, and rs2070069 loci are located in the noncoding region of the genes. The rs3087353 and rs2070068 loci are situated within exons, yet nucleotide substitutions within them fail to result in amino acid substitutions. The alleles listed in Table 3 are linked in both indigenous Siberians and other East Asian populations, including Japanese, Koreans, and Vietnamese (Tables 3 and 4). In individuals from Siberia, all these alleles are known to be present in the rs4988235-CC genotype, which is associated with primary hypolactasia. The rs79023654 locus of the LCT gene is located at a distance of ~29.7 thousand nucleotide pairs from the rs4988285 locus of the MCM6 gene. The polymorphic loci within the MCM6 gene are located at a distance of ~26.5 thousand nucleotide pairs from each other. Furthermore, the rs4988285 and rs2070069 loci are located in the vicinity of the enhancer that regulates the activity of the LCT gene. This suggests the potential functional significance of the identified haplotype’s polymorphic variants.

The analysis of the dbSNP data (https://www.ncbi.nlm. nih.gov/snp/) indicated that the rs79023654-A, rs4988285-A, rs2070069-G, and rs3087353-T variants were characteristic of the East Asian population and were observed with a low frequency (approximately 1 %) in the South Asian populations (Table 4). However, the fifth allele from this group, rs2070068- A, was detected with a relatively high frequency (24.7 %) in African populations (Table 4). From this distribution, it can be concluded that the East Asian haplotype rs79023654-A, rs4988285-A, rs2070069-G, and rs3087353-T was formed on the basis of ancestral (African) haplotypes, which were characterized by the rs2070068-A variant. However, an analysis of paleogenomic data (AADR database) revealed that the rs2070068-A variant emerged in Africa at a later point in time than in Eurasia. The earliest documented occurrence of this allele in Africa is associated with the northern region of the continent (in the territory of Morocco) at approximately 14.5 kya. Subsequent cases were identified at approximately 9 kya and later. However, it became evident that in Eurasia, this variant of MCM6 gene polymorphism was observed in both Denisovans and Neanderthals (individuals who lived between ~40 and 110 kya), as well as in numerous most ancient representatives of Homo sapiens in Europe and East Asia (aged between ~34 and 44 kya)

**Table 4.**
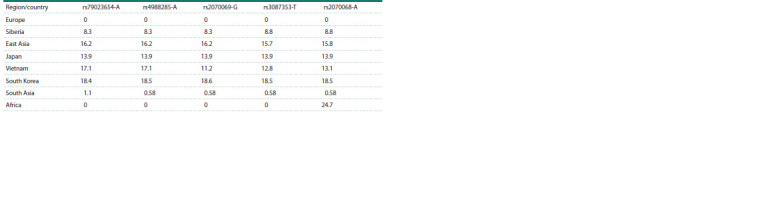
Frequency (in %) of variants rs79023654-A, rs4988285-A, rs2070069-G, rs3087353-T and rs2070068-A in world populations Notе. Population frequencies are given according to the dbSNP database; for Siberian populations, according to Pagani et al. (2016).

Further analysis of paleogenomic sequence databases (Denisova Variants Track Settings; https://genome.ucsc.edu/ cgi-bin/hgTrackUi?db=hg19&g=dhcVcfDenisovaPinky) revealed that the MCM6 haplotype rs4988285-A, rs2070069-G, rs3087353-T, rs2070068-A was common among Denisovans and Neanderthals. The rs79023654 locus of the LCT gene fell into a sequencing region with low coverage. Hence the presence of polymorphism at this locus in Denisovans and Neanderthals remains uncertain

The results obtained thus suggest that the MCM6 haplotype detected in the population of East Asia (and, to a much lesser extent, South Asia) is archaic. It is possible that this haplotype was inherited from the common ancestor of H. sapiens, Neanderthals, and Denisovans (approximately 600 kya, at the time of the divergence of the ancestor of H. sapiens from the ancestors of Neanderthals and Denisovans, as reported by H. Zeberg et al. (2024)). Alternatively, it may have been acquired as a result of hybridization with Neanderthals or Denisovans. Given the distribution of the archaic haplotype in East Asia, it seems more probable that introgression from Denisovans occurred. It has been demonstrated that Neanderthals and Denisovans also exchanged genes – for example, approximately 80–90 kya in southern Siberia (Slon et al., 2018). Consequently, the hypothesis that polymorphic variants were transferred from Denisovans to Neanderthals is also a plausible one

In recent years, there has been a considerable amount of work done to catalogue archaic variants of genetic polymorphisms that have been identified in the gene pool of modern humans (https://bioinf.eva.mpg.de/catalogbrowser), but the incompleteness of this type of information may be dependent on the extent to which populations have been studied (Zeberg et al., 2024). It seems likely that this database will become much more comprehensive as genomic research continues to expand geographically. There are already some interesting findings of rare ancestral polymorphism variants in widely separated populations – for example, identical alleles of a number of genes in the South African Khoisan and the Philippine Aeta (Zeberg et al., 2024).

There is a great deal of information about the genetic variants that modern humans have inherited from Neanderthals. In particular, there is much to be gained from an understanding of the advantages that humans have gained from admixture, in terms of metabolism, sensory function (especially pain perception), immunity (including SARS-CoV-2), and the expression of some genes (Telis et al., 2020; Zeberg et al., 2020; Pairo-Castineira et al., 2021; Haeggström et al., 2022; Zeberg et al., 2024).

Much less is known about the functional manifestations of Denisovan genetic influence. The main examples of such influence are related to adaptation to high altitude and cold conditions. For instance, a ~33,000 bp fragment of Denisovan DNA has been found in Tibetans that encodes the hypoxia-inducible transcription factor EPAS1, which is involved in adaptation to low oxygen levels (Zhang et al., 2021). In Greenland Eskimos, a ~28,000 bp fragment of Denisovian DNA containing the WARS and TBX15 genes has been identified with high frequency – it is believed that these polymorphic variants may play a role in the adaptation of Arctic indigenous peoples to low temperatures (Racimo et al., 2017). It seems plausible to suggest that the archaic haplotype of the MCM6 gene found in East Asian populations may be used to implement a specific programme for regulating the enzymatic activity of lactase, which is still relevant today. Further studies are needed to gain a deeper understanding of the specific role of this haplotype in regulating lactase activity. These studies should consider a range of factors, including medical genetics, biochemical and physiological aspects.

## Conclusion

Thus, the results of the review of the data on the variability of the LCT and MCM6 genes indicate that from ancient times the indigenous populations of Siberia have been characterized by a low frequency of the rs4988235-T variant, which may contribute to the enhancement of the enzymatic activity of lactase. A certain increase in the frequency of this allele over time in the populations of Central Asia and Southwestern Siberia is associated with the eastward expansion of the ancient populations of the Eastern European steppes starting from the Bronze Age (Heyer et al., 2011; Pilipenko et al., 2016; Segurel et al., 2020). However, it seems that the rs4988235-T variant did not reach high frequencies in Central Asian populations, in contrast to Europe. This may suggest that there is no significant selective pressure on this variant of polymorphism in Central Asian populations (Segurel et al., 2020). It is still unclear why different groups of East Asian populations that traditionally consume dairy products have not developed specific variants of genetic polymorphisms for lactose metabolism. One possible explanation is the hypothesis of cultural adaptation of Central Asian populations, including the development of a culture using bacteria to digest lactose during fermentation, which may have contributed to the establishment of specific microflora in the gut (Segurel et al., 2020)

It is also worth noting that some epigenetic mechanisms (mainly DNA methylation) may also be involved in regulating the expression of lactose metabolism genes (Labrie et al., 2016). It has also been suggested that the type of DNA methylation in the enhancer and promoter regions of the LCT gene may be a useful indicator of lactase phenotypes, and it appears that epigenetic modifications may play an important role in the regulation of lactase deficiency (Leseva et al., 2018). Thus, both genetic and epigenetic approaches should be used to investigate the functional significance of polymorphic variants potentially associated with LP, including archaic genetic variants, which the present study has shown to still have some prevalence in human populations.

## Conflict of interest

The authors declare no conflict of interest.
